# Nerve Regeneration and Gait Function Recovery with Implantation of Glucose/Mannose Conduits Using a Rat Model: Efficacy of Glucose/Mannose as a New Neurological Guidance Material

**DOI:** 10.3390/bioengineering11020157

**Published:** 2024-02-04

**Authors:** Osamu Yamamoto, Risa Saito, Yuta Ohseki, Asami Hoshino

**Affiliations:** Graduate School of Science and Engineering, Yamagata University, 4-3-16 Jonan, Yonezawa 992-8510, Yamagata, Japan; orisaorisa0125@docomo.ne.jp (R.S.); kenkyuuyou0511@gmail.com (Y.O.); asami07018347@gmail.com (A.H.)

**Keywords:** nerve guidance materials, motor function, glucose/mannose, mechanical property, regenerative nerve, hydrophilicity

## Abstract

Therapy with clinical nerve guidance conduits often causes functional incompleteness in patients. With the aim of better therapeutic efficacy, nerve regeneration and gait function were investigated in this study using a novel nerve guidance conduit consisting of glucose/mannose. The glucose/mannose nerve guidance conduits were prepared by filling the conduits with the glucose/mannose aqueous solutions for different kinematic viscosity, which were applied to sciatic nerve defects (6 mm gap) in a rat model. The nerve regeneration effect and the gait function recovery with the fabricated nerve guidance conduits were examined. From the results of the XRD measurement, the glucose/mannose conduits were identified as crystal structures of cellulose type II. Young’s modulus and the maximum tensile strength of the crystalline glucose/mannose conduits demonstrated good strength and softness for the human nerve. Above 4 weeks postoperative, macroscopic observation revealed that the nerve was regenerated in the defective area. In various staining results of the nerve tissue removed at 4 weeks postoperative, myelinated nerves contributing to gait function could not be observed in the proximal and distal sites to the central nerve. At 8–12 weeks postoperative, myelinated nerves were found at the proximal and distal sites in hematoxylin/eosin staining. Glia cells were confirmed by phosphotungstic acid–hematoxylin staining. Continuous nerve fibers were observed clearly in the sections of the regenerated nerves towards the longitudinal direction at 12 weeks postoperative. The angle between the metatarsophalangeal joint and the ground plane was approximately 93° in intact rats. At 4 weeks postoperative, walking was not possible, but at 8 weeks postoperative, the rats were able to walk, with an angle of 53°. At 12 weeks postoperative, the angle increased further, reaching 65°, confirming that the rats were able to walk more quickly than at 8 weeks postoperative. These results demonstrated that gait function in rats treated with glucose/mannose nerve guidance conduits was rapidly recovered after 8 weeks postoperative. The glucose/mannose nerve guidance conduit could be applied as a new promising candidate material for peripheral nerve regeneration.

## 1. Introduction

Central nerves have poor regenerative ability, but peripheral nerves have high regenerative ability. When peripheral nerves are injured, their axons regenerate after Wallerian degeneration, and Schwann cells play a central role in this process. If the peripheral nerves are severed without a nerve gap at the injury site, nerve regeneration may be impossible. In this case, the direct suturing of a nerve stump using a microscope would be chosen as the standard treatment in plastic and reconstructive surgery. If the nerve gap has a relatively long distance to be closed with direct sutures, the normal nerve may be stretched and damaged. The nerve gap should be filled to restore nerve continuity. In such cases, the conventional treatment is autologous nerve transplantation [1], but neuropathy at the nerve harvesting site is a problem. Mackinnon et al. reported that 20–40% recovered very well after direct nerve transplantation, but few injuries recovered completely [2]. Additionally, the locations and lengths of nerves that can be harvested are limited. Therefore, nerve guidance conduits are developed as an alternative to nerve grafting, which can be implanted at the site of a nerve gap to serve as a scaffold for nerve regeneration. Nerve guidance conduits have been used primarily in clinical treatment to achieve regeneration without sacrificing functional nerves [3].

Recent studies have reported that nerve guidance conduits provide better outcomes than nerve anastomoses when peripheral nerve transection results in a gap [4,5,6,7,8]. Most nerve guidance conduits in clinical and research reports have consisted of hollow conduits filled with collagen [9,10,11,12,13]. In contrast, there are many kinds of hollow conduits, including collagen, silicon, polyethylene, polyglycolic acid (PGA), chitosan, etc. [14,15,16,17,18,19]. Several nerve guidance conduits mentioned above are in clinical trials, and a few guidance conduits have already been applied to patients in Japan. However, the results of treatments using nerve guidance conduits are often incomplete, as expressed in symptoms such as decreased sensation, decreased motor function, and pain compared to before the nerve damage. These factors can lead to problems in quality of life, work, leisure, and social life associated with functional impairment. The reason for the incompleteness of nerve regeneration may be due to the shape of the severed nerve tip and the inhibition of axonal extension. Thus, the present work focused on the mechanism of axonal extension.

Axons have been known to elongate by the amoeboid movement of the motile growth cone at the severed nerve tip, which is triggered by actin fibers and motor molecules present in the growth cone [20]. The energy for this movement is provided by adenosine triphosphate (ATP). Based on the fundamental knowledge that energy is led by the glycolytic system of the saccharides, glucose/mannose, one of the saccharides, was selected as a new nerve guidance material. However, there are no studies on nerve guidance conduits fabricated by glucose/mannose.

In the present work, nerve guidance conduits were fabricated from a glucose/mannose aqueous solution. To clarify the effectiveness of the glucose/mannose nerve guidance conduits on nerve regeneration, the material characteristics of the fabricated conduits were examined, including the mechanical and surface properties of the hollow conduits, and the viscosity of the solution inside the conduits. Further, nerve regeneration and gait function recovery with the implantation of the fabricated glucose/mannose nerve guidance conduits were evaluated using the defect model of the rat’s sciatic nerve.

## 2. Materials and Methods

### 2.1. Preparation of Glucose/Mannose Viscous Solution as a Filler

Glucose/mannose powder (PROPOL^®^A) with an average molar ratio (glucose/mannose) of about 0.1 was procured from SHIMIZU Chemical Co. (Hiroshima, Japan), of which the inspection results for safety standard values to humans are listed in Table 1. The powder was added to distilled water at a concentration of either 10 or 14 g/L; then, the glucose/mannose aqueous solution was stirred at 26 °C for 90 min. After stirring, the as-prepared solution was sterilized by an autoclave (LBS-245; Tomy Seiko Co. Ltd., Tokyo, Japan) at 121 °C for 20 min, and the resulting viscous solution was used as a filler for hollow nerve guidance conduits.

The static and kinematic viscosities of the glucose/mannose viscous solution as a filler were examined by tuning fork vibration viscometer (SV-10A, A&D Instrument Inc., Tokyo, Japan) and rotational viscometer (TV-20, Toki Sangyo Co. Ltd., Tokyo, Japan), respectively. In these viscosity measurements, ten batches of glucose/mannose solutions were measured using 50 mL for static viscosity and 200 mL for dynamic viscosity measurements.

### 2.2. Fabrication of Hollow Glucose/Mannose Conduits

**Figure 1:**
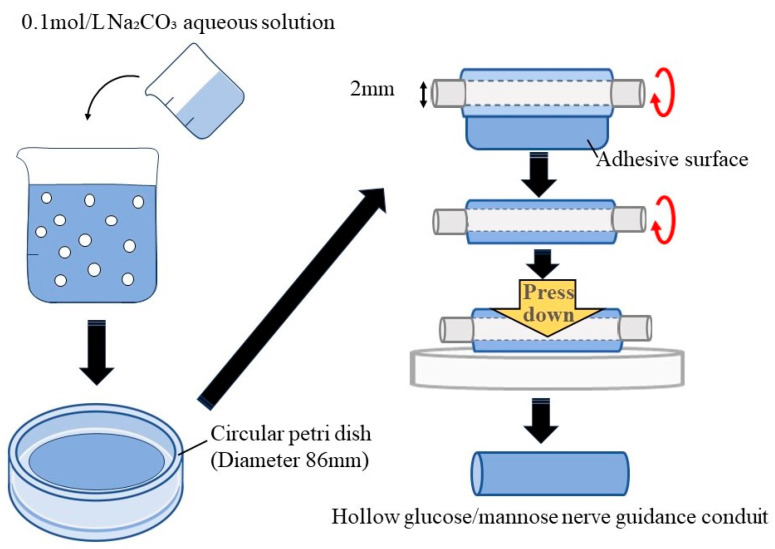
Schematic illustration of the fabrication process of hollow glucose/mannose conduits.

Figure 1 shows the fabrication process of hollow glucose/mannose conduits. Firstly, 0.1 mol/L sodium carbonate (Na_2_CO_3_, Nacalai Tesque Inc. Kyoto, Japan) aqueous solution was prepared prior to the fabrication of the glucose/mannose conduits, and 0.5 g of glucose/mannose powder was dissolved in 50 mL of distilled water. The glucose/mannose aqueous solution was then stirred with a magnetic stirrer for 90 min at 26 °C. Then, 20 mL of as-prepared Na_2_CO_3_ aqueous solution was added to the glucose/mannose aqueous solution. The mixed solution was further stirred for 3 min. Additionally, 35 mL of the mixed solution was cast onto a circular petri dish with a diameter of 86 mm and dried for 5 days at 26 °C. The dry glucose/mannose films with a thickness of approximately 0.1 mm were washed with distilled water several times until the films showed pH 7. The neutralized films were wrapped one time around a titanium rod with a diameter of 2 mm. To adhere to the wrapped films, the mixed solution already prepared was applied to the adhesive surface; then, the adhesive area was pressurized to form hollow conduits. The hollow conduits with an inner diameter of 2 mm and a thickness of approximately 0.1 mm thus prepared were autoclaved and used as the conduit of nerve guidance. Dimension measurement of the hollow guidance conduits was conducted using a digital micrometer with a wide range (Mitutoyo, Kanagawa, Japan).

### 2.3. Characterization of Hollow Glucose/Mannose Conduits

Nerve guidance materials should be designed with the mechanical and biochemical cues necessary for nerve regeneration [21]. As an evaluation commensurate with the design, surface and mechanical properties are essential to examine the hollow nerve guidance conduits consisting of glucose/mannose. The crystal structure on the surface of hollow glucose/mannose conduits was analyzed using a thin film X-ray diffraction meter (TF-XRD, UltimaIV, Rigaku, Tokyo, Japan), in which the measurement conditions were voltage: 40 kV, current: 40 mA, scanning speed: 2°/min, and step width: 0.02. The detected XRD diffraction peaks were normalized by correcting the 2θ angle using standard silicon. The surface wettability of hollow glucose/mannose conduits was measured by the contact angle of 6 μL H_2_O dropped from a height of 10 mm onto the conduit surface. The surface roughness on the conduits was also determined using software (VK-Analyzer) installed on a confocal laser microscope (VK 9700 Violet Laser, Keyence, Osaka, Japan). Twenty measurement points were randomly selected from the surface of the conduits, and the results were expressed as the average surface roughness, *Ra*.

The mechanical properties of the hollow nerve guidance conduits consisting of glucose/mannose were determined by the tensile test of the hollow conduits using an autograph with a 10 kN load cell (AGS-J, Shimadzu Co., Kyoto, Japan). The load measurement accuracy was −0.5%. The fabricated hollow conduits were immersed in distilled water for 10 min and then placed so that the distance between the fixtures of the autograph was 14 mm. The tensile test continued until the hollow conduits broke under a tensile speed of 1.0 mm/min and a sampling time interval of 50 ms. The detected test force was divided by the cross-sectional area (0.62 ± 0.11 mm^2^) of the hollow conduits, and then a stress–strain curve was drawn by dividing the length of the hollow conduits extended by the distance between the fixtures. An approximated stress–strain curve was created from the least squares method using a Kaleida Graph (Ver. 5.0.4, HULINKS Inc., Tokyo, Japan). Young’s modulus was determined from the slope of the approximated curve. The maximum tensile strength for the hollow conduits was defined as the value of the breaking point during the tensile test. The maximum tensile strength and Young’s modulus values were expressed as an average of 10 measurements.

### 2.4. In Vivo Evaluation of Nerve Regeneration Using Glucose/Mannose-Derived Nerve Guidance Conduits

#### 2.4.1. Animal Experiment

##### Preparation for Animal Surgery

The glucose/mannose-derived hollow conduits and viscous fillers were sterilized in an autoclave before application to rats. All surgical tools, such as scalpels, forceps, and scissors, were also sterilized in an autoclave. In addition, suture threads and needles were sterilized with an ethylene oxide gas.

Regarding the care and treatment of experimental animals, the animal experiment protocol in this study was approved by the Yamagata University (Yamagata Prefecture) Animal Committee. Four rats aged 11–14 weeks (male, Crl:SD, Japan SLC, Inc., Shizuoka, Japan), weighing 290–370 g, were used in each postoperative period and glucose/mannose concentration. A triple anesthetic mixture was employed in this study. The amounts of the three anesthetics were 0.15 mg/Kg for medetomidine hydrochloride (Nippon Zenyaku Kogyo Co., Ltd., Fukushima, Japan), 2.0 mg/Kg for midazolam (Astellas Pharma Inc., Tokyo, Japan), and 2.5 mg/Kg for butorphanol tartrate (Meiji Seika Pharma Co., Ltd., Tokyo, Japan) based on the rat body weight (kg). To sedate the rats before administration of a triple anesthetic mixture, 3% sevoflurane (Maruishi Pharmaceutical Co., Ltd., Osaka, Japan) was used at a flow rate of 3 mL/min and voluntarily inhaled into the rats through the nose. After adequate sedation, the rats were anesthetized by intramuscular administration of a triple anesthetic mixture. The sciatic nerve can be reached from the lateral side of the rat thigh. Therefore, after removing the hair on the thigh with clippers, the remaining hair was removed completely using hair removal cream. Then, the thigh was disinfected with 7% povidone-iodine.

##### Application of Guidance Conduits through Animal Surgery

**Figure 2:**
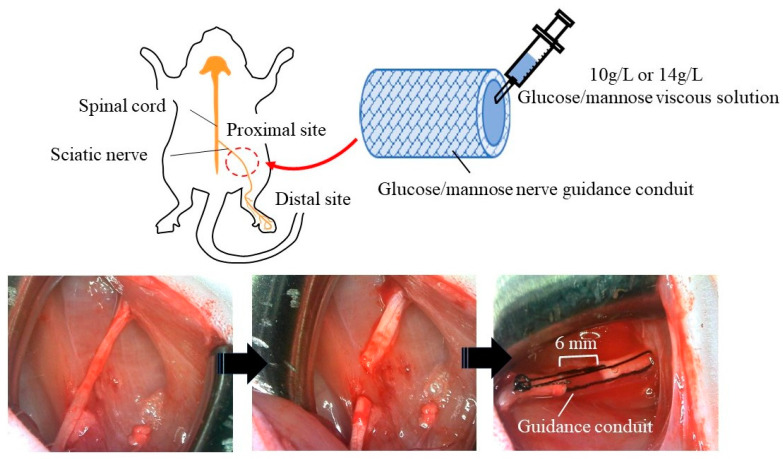
Application process for sciatic nerve of the glucose/mannose nerve guidance conduits fabricated.

Prior to the application of the hollow nerve guidance conduits and fillers consisting of glucose/mannose to rats, a cytotoxicity test was conducted using L929 cells according to JIS (T 0993) and ISO 10993standards. The results showed that the materials used in this study were not cytotoxic in vitro. Figure 2 shows photographs of the application of guidance conduits. The sciatic nerve was exposed by incising the skin of the thigh and then peeling away between the subcutaneous muscles and then severed after careful removal of the surrounding tissue of the epineurium. Both ends of the severed nerve were inserted into the hollow guidance conduits, and the epineurium and conduit were sutured using 6-0 sutures to create a 6 mm nerve gap. The gap inside the hollow conduits was filled with the previously prepared glucose/mannose viscous solutions. 

### 2.5. Histological Evaluation

Postoperative rats were kept under spontaneous feeding and euthanized at 4–12 weeks. Regenerated nerves were collected, including nervous tissue from near the central nerve to near the knee joint. The regenerated sciatic nerves were fixed in a 5% formaldehyde–phosphate buffer and embedded in paraffin blocks for histological staining. The paraffin blocks were then cut into thin specimens with a thickness of 6 μm longitudinally and vertically to the long axis of the nerve. In particular, the presence of glial cells and myelinated nerves in the regenerated nerves needs to be confirmed. This is because glial cells play an important role in the formation of neuronal circuits as well as in supplying nutrients to the nerve cells. Furthermore, myelinated nerves control motor and sensory functions. Therefore, tissue specimens were stained by using hematoxylin–eosin (HE) for myelinated nerves and phosphotungstic–hematoxylin (PTAH) for glial cells. All staining solutions were purchased from Muto Chemical Co., Ltd. (Tokyo, Japan).

### 2.6. Gait Function

**Figure 3:**
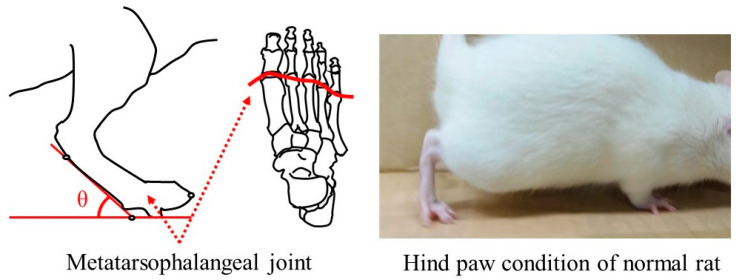
Measurement of the angle between the metatarsophalangeal joint and the ground at spontaneous walking of the rat.

In normal rats, the metatarsophalangeal joints are perpendicular to the ground at an angle greater than 90° with the toes raised at the walk, as shown in Figure 3. This gait status indicates that the nerve transmission on gait function is normal in the lower limb region from the thigh to the toes. The rats at 4–12 weeks postoperative were placed on a path with a 200 mm width and a 1500 mm distance, and then spontaneous gait was recorded in video mode on a digital camera (COOLPIX A1000, Nikon Co., Tokyo, Japan). The angle of the metatarsophalangeal joint to the ground during walking was determined by focusing on the lower limbs of the photographed rats. The measured angle was defined as a guideline for recovery of gait function.

### 2.7. Statistical Analysis

All data were expressed as mean ± standard deviation (SD). Considering the importance of statistical analysis, the obtained results were analyzed using one-way analysis of variance (ANOVA) and Tukey’s post hoc test for multiple comparisons. Statistical significance (*p* < 0.05) was expressed at the 5% significance level.

## 3. Results

### 3.1. Viscosity of Glucose/Mannose Aqueous Solution

**Figure 4:**
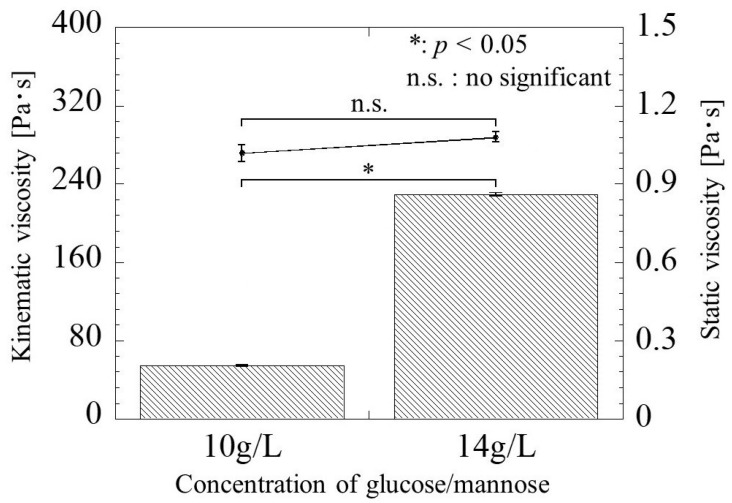
The static and kinematic viscosity versus glucose/mannose concentration. The line graph for the static viscosity and the bar graph for the kinematic viscosity represent the value of the static viscosity on the right and the kinematic viscosity values on the left.

Figure 4 shows the static and kinematic viscosity versus glucose/mannose concentration at 26 °C. The static viscosity at the concentration of 10 and 14 g/L was 1.02 ± 0.03 Pa·s and 1.08 ± 0.02 Pa·s, respectively, which increased slightly by increasing the concentration of glucose/mannose in distilled water. However, there was no significant difference in static viscosity between the two concentrations. On the other hand, the kinematic viscosity was 54.10 ± 0.70 Pa·s at a concentration of 10 g/L and significantly increased to 229.40 ± 1.72 Pa·s at a concentration of 14 g/L. The behavior of viscosity as a function of glucose/mannose concentration was found to differ greatly between static and kinematic viscosity.

### 3.2. Crystal Structure of Hollow Glucose/Mannose Conduits

**Figure 5:**
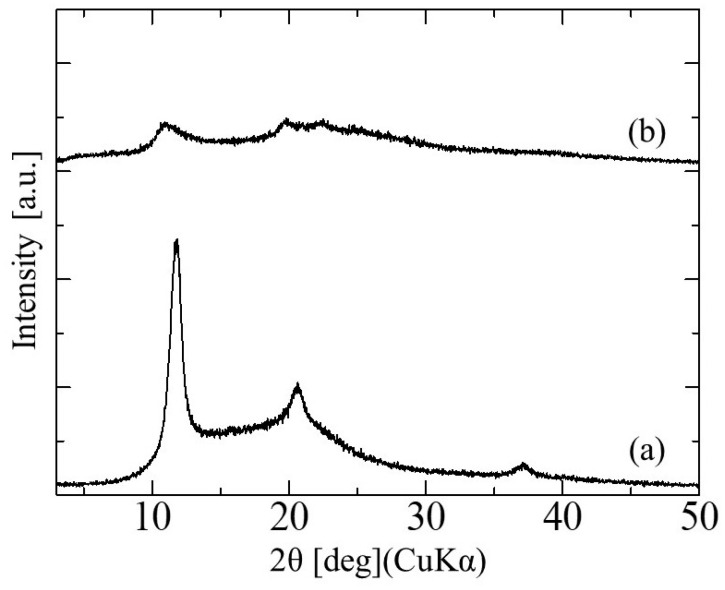
The X-ray diffraction patterns of glucose/mannose guidance conduits (**a**) before and (**b**) after immersion in distilled water for 10 min.

The XRD patterns of the glucose/mannose conduits before and after immersion in distilled water for 10 min are shown in Figure 5. The dry conduits, i.e., before immersion, demonstrated three diffraction peaks, which corresponded to the typical pattern of cellulose type II with a layered structure [22]. The interlayer distance determined from the diffraction peak at 2θ = 11.84° was 0.747 nm. These peaks detected were broadened after immersion for 10 min, of which the intensity decreased. Moreover, the diffraction peak at 11.84° in the 2θ shifted to the low angle side to 11.17° in the wet conduits immersed in distilled water. The interlayer distance based on this shift was calculated to be 0.792 nm.

### 3.3. Surface and Mechanical Properties of Hollow Glucose/Mannose Conduits

#### 3.3.1. Surface Roughness

**Figure 6:**
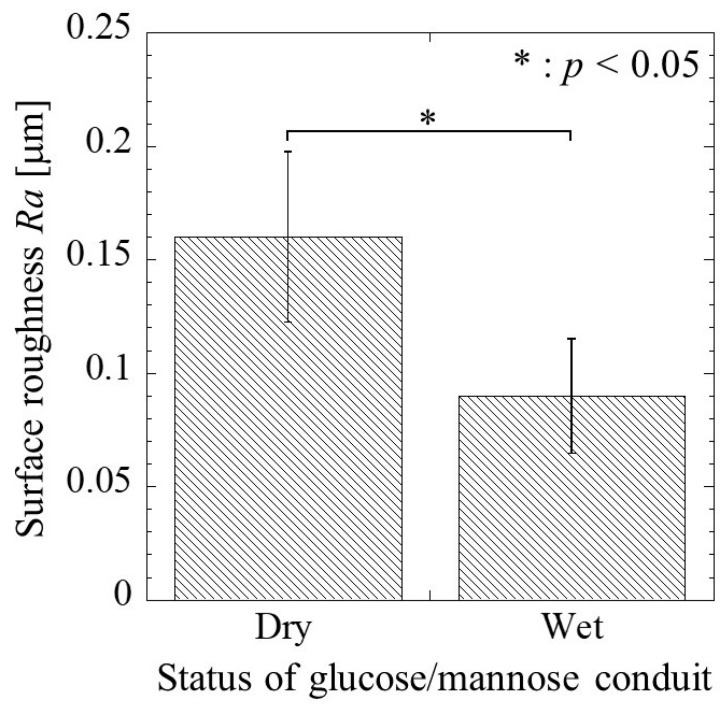
The surface roughness, *Ra*, of the glucose/mannose guidance conduits before (dry) and after (wet) immersion in distilled water for 10 min.

Figure 6 shows the surface roughness of the glucose/mannose conduits before and after immersion in distilled water for 10 min. The surface roughness before immersion, the dry conduits, yielded 0.16 ± 0.04 mm as the arithmetic mean roughness, *Ra*. In the case of the wet conduits after immersion, the surface roughness exhibited a value of 0.09 ± 0.03 mm. That is, the surface roughness of the wet conduit was found to significantly reduce compared to that of the dry conduit.

#### 3.3.2. Wettability

**Figure 7:**
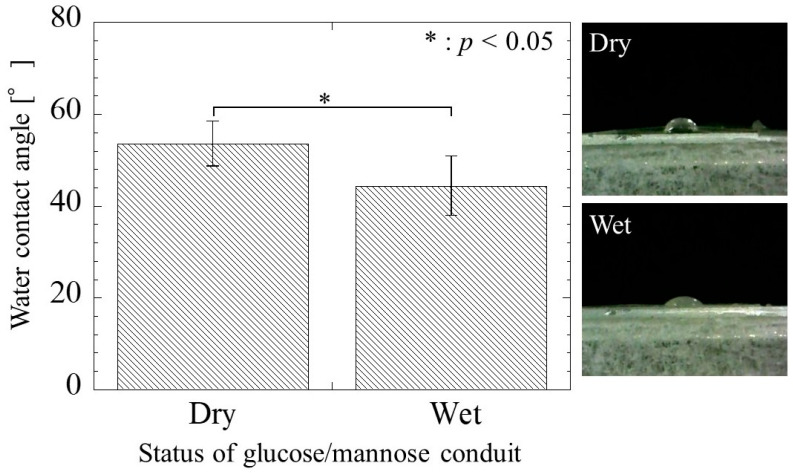
The water contact angle of the glucose/mannose guidance conduits before (dry) and after (wet) immersion in distilled water for 10 min.

The contact angle of the glucose/mannose conduits before and after immersion in distilled water for 10 min is shown in Figure 7. The contact angle of the dry conduits before immersion was a value of 53.52 ± 4.90°, whereas the wet conduits after immersion exhibited a contact angle of 44.36 ± 6.47° smaller than in the dry conduits. There was a significant difference between the dry and wet conduits. As a result, the wettability of the wet conduits was found to be higher than that of the dry conduits.

#### 3.3.3. Mechanical Property

**Figure 8:**
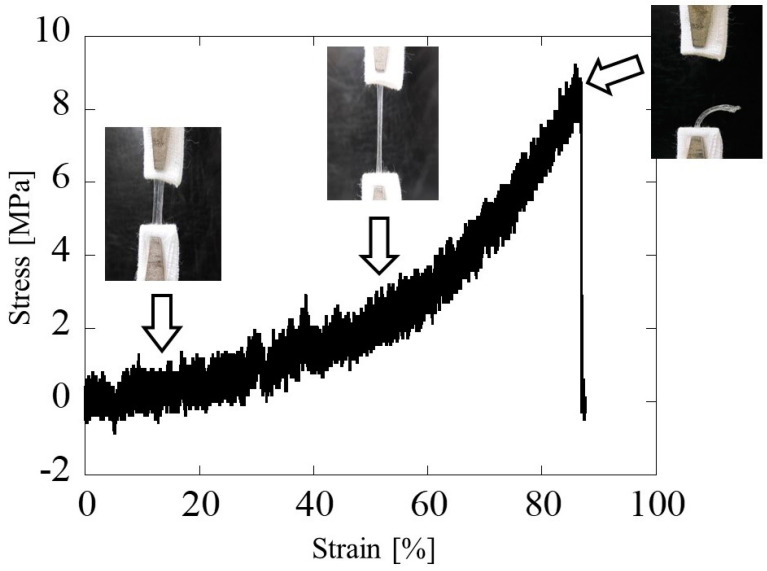
Typical stress–strain curve and tensile extension photos of the glucose/mannose guidance conduits immersed in distilled water for 10 min.

Figure 8 shows a typical stress–strain curve in the hollow glucose/mannose conduits immersed in distilled water for 10 min. The stress–strain curve showed a linear increase in stress with increasing strain, with a slight bend at 55% strain and a change in the slope of the stress–strain curve. The Young’s modulus in the region below and above 55% strain were determined to be 8.80 ± 0.59 and 16.92 ± 3.10 MPa, respectively. The maximum tensile strength was found to be 9.23 ± 0.91 MPa.

### 3.4. Evaluation of Regenerated Nerve

**Figure 9:**
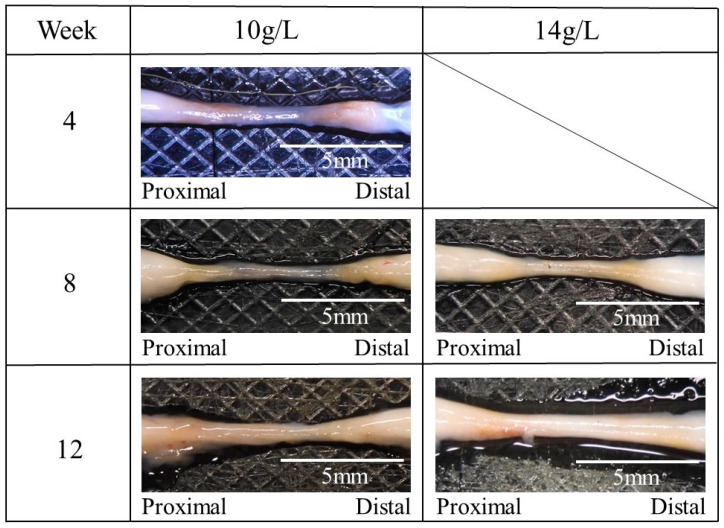
The macroscopic photos of the regenerated nerve using the glucose/mannose nerve guidance conduits with a filler of the glucose/mannose at a concentration of 10 and 14 g/L.

The regenerated sciatic nerve inside the nerve guidance conduits was removed at 4–12 weeks postoperative. After this removal, residual transparent guidance conduits without a filler were observed. Figure 9 shows the macroscopic observations of the nerve regenerated using the nerve guidance conduits with a filler of the glucose/mannose at a concentration of 10 and 14 g/L. The regenerated nerve was pale black at 4 weeks postoperative but changed from pale black to white as the postoperative period progressed. 

**Figure 10:**
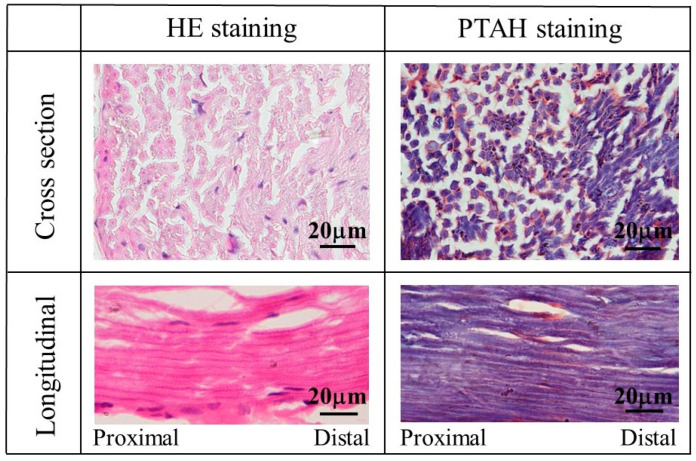
The images of HE- and PTAH-stained tissue of the autologous sciatic nerve.

To compare the tissue of the regenerated sciatic nerve, HE- and PTAH-stained images of the tissue on the autologous sciatic nerve are shown in Figure 10. In these stained images, many myelinated nerves and glia cells could be observed in HE and PTAH staining, respectively, at nerve cross-sections. In longitudinal staining images along the length of the nerve, nerve fibers with myelinated nerves and glial cells were observed as continuous tissue without interruption. 

**Figure 11:**
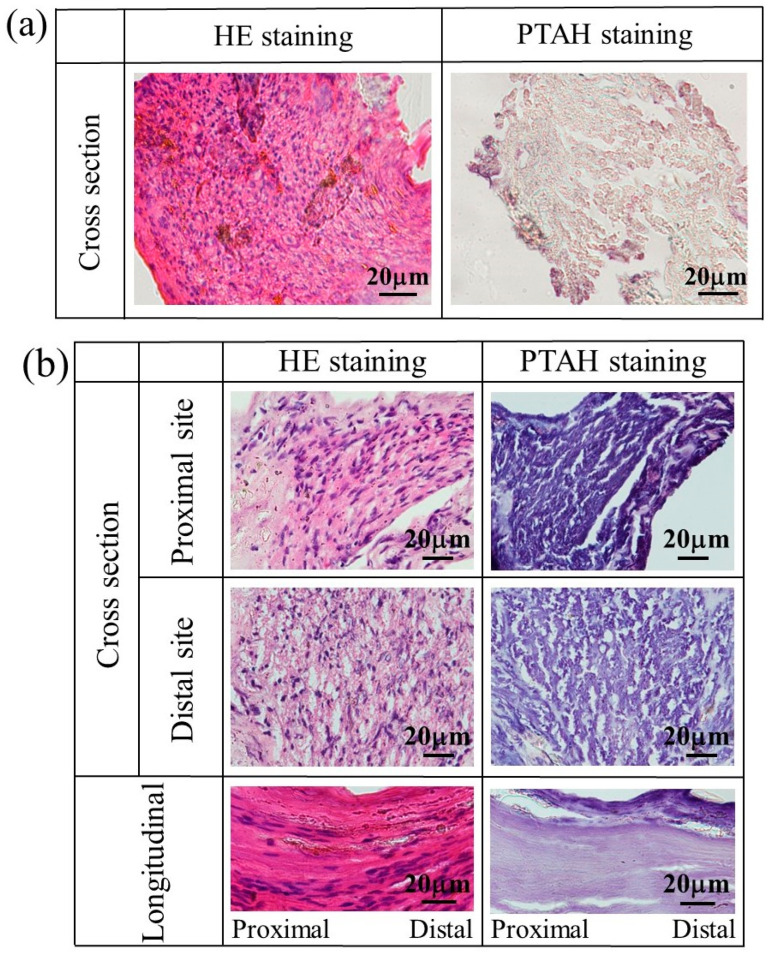

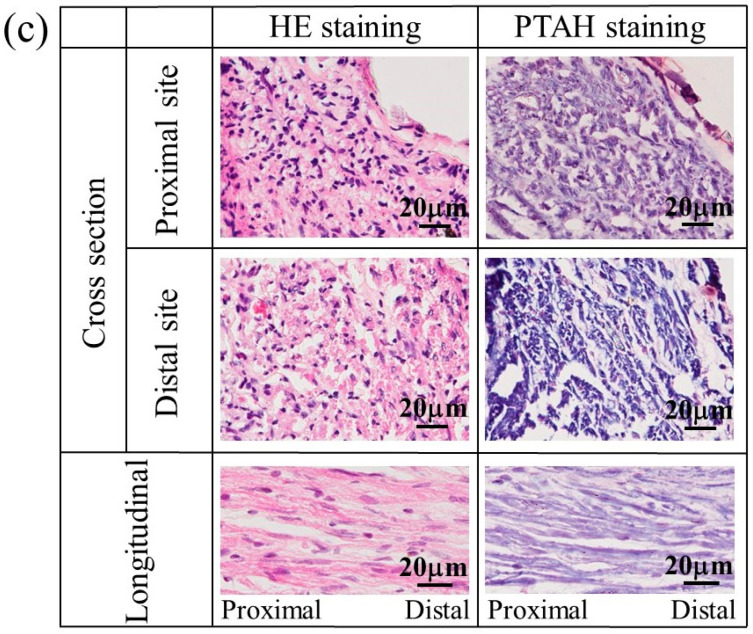
The stained images of the tissue regenerated at (**a**) 4, (**b**) 8, and (**c**) 12 weeks postoperative using a glucose/mannose filler at a concentration of 10 g/L.

Figure 11 shows the stained images of the tissue regenerated at 4–12 weeks postoperative using a glucose/mannose filler at a concentration of 10 g/L. As shown in Figure 11a, quite unlike autologous nerves, myelinated nerves, and glial cells were slightly observed at 4 weeks, and strong inflammatory cell infiltration was revealed in HE staining. At 8 weeks (see Figure 11b), the cross-sectional HE-stained images showed relatively mild inflammatory cell infiltration at distal and proximal sites to the central nerve, while myelinated nerves and glial cells could be observed. In the longitudinally stained images, the continuous fibrous issue could be confirmed from HE- and PTAH-stained images. Myelinated nerves and glial cells within the regenerated tissue at 12 weeks were more clearly observed in the cross-sectional stained images (see Figure 11c) compared to the tissue regenerated at week 8. The continuity of nerve fibers seen in the longitudinal stained images was also more evident at 12 weeks than at 8 weeks.

**Figure 12:**
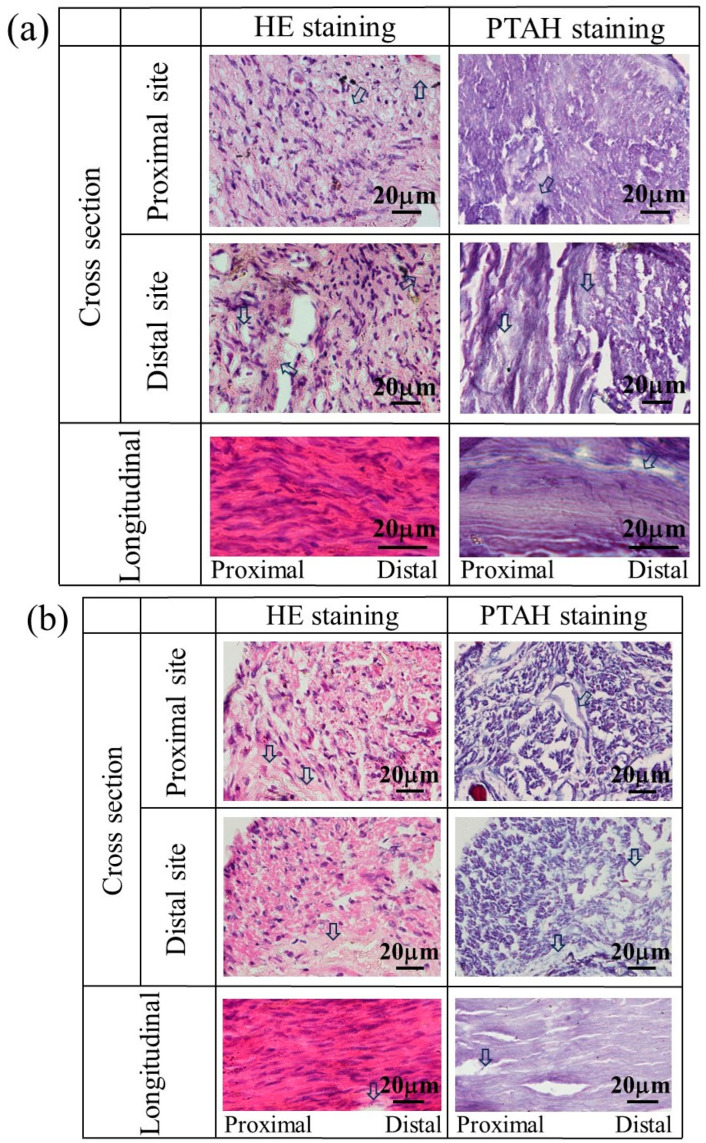
The stained images of the tissue regenerated at (**a**) 8 and (**b**) 12 weeks postoperative using a glucose/mannose filler at a concentration of 14 g/L. Arrows represent residual glucose/mannose.

The nerves regenerated using a glucose/mannose filler at a concentration of 14 g/L were also stained to investigate the influence of the kinematic viscosity of glucose/mannose on the generation of myelinated nerves and glial cells. Figure 12 shows the stained images of the tissue regenerated at 8 and 12 weeks. At 8 weeks (see Figure 12a), HE- and PTAH-stained cross-sectional images demonstrated not only myelinated nerves and glial cells at proximal and distal sites but also unstained portions presumed to be residual glucose/mannose. The status of inflammatory cell infiltration and consecutive nerve fibers was almost the same as the regenerated nerves that occurred in 8 weeks using a glucose/mannose filler at a concentration of 10 g/L. As shown in Figure 12b, the nerves regenerated at 12 weeks were similar to those at 8 weeks using a glucose/mannose filler at a concentration of 10 g/L, as related to the development of myelinated nerves and glial cells, and further the continuity of nerve fiber. Unstained portions were also observed at 12 weeks, probably due to residual glucose/mannose. The staining results of the regenerative nerves revealed that glucose/mannose remained in the regenerative nerves as the kinematic viscosity of glucose/mannose increased.

### 3.5. Gait Function of Rats with the Postoperative Period

**Figure 13:**
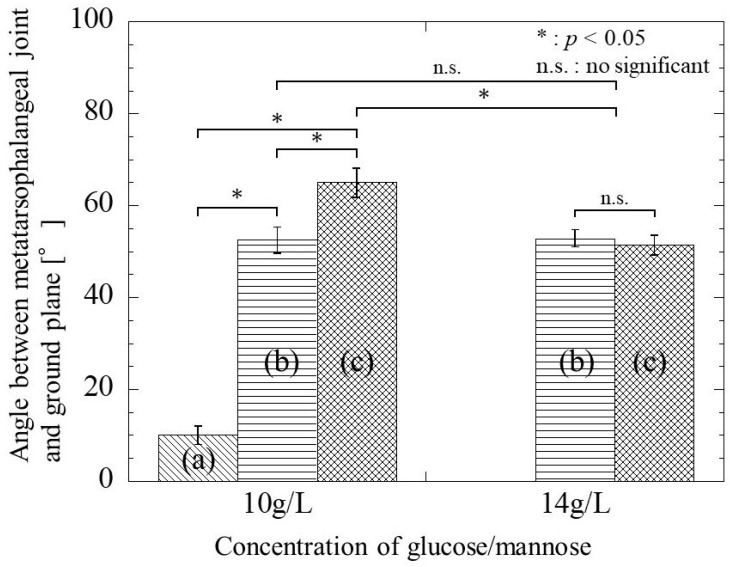
The angle between the metatarsophalangeal joint and the ground plane during natural walking of postoperative rats at (**a**) 4 weeks, (**b**) 8 weeks, and (**c**) 12 weeks.

Normal rats walked with the toes raised, and the metatarsophalangeal joint was 93.34 ± 1.87° to the ground, as shown in Figure 3. Figure 13 shows the angle between the metatarsophalangeal joint and ground plane when the postoperative rats walk naturally. In the case that glucose/mannose at a concentration of 10 g/L was used as a filler of hollow nerve guidance conduits, the angle was 9.99 ± 2.06° at 4 weeks postoperative. In other words, this means that rats had difficulty walking. The angle at 8 weeks postoperative significantly increased to 52.52 ± 2.87° compared to 4 weeks postoperative. With the increase in the postoperative period, the angle reached 64.98 ± 3.20° at 12 weeks, with a significant difference at 4 and 8 weeks. On the other hand, using a glucose/mannose filler at a concentration of 14 g/L demonstrated that the angle between the metatarsophalangeal joint and ground plane was 52.86 ± 1.85° at 8 weeks and 51.39 ± 2.16° at 12 weeks. No significant differences were found between 8 and 12 weeks in this concentration of glucose/mannose. Furthermore, these values were similar without significant difference to 8 weeks in a glucose/mannose filler at a concentration of 10 g/L. From these results mentioned above, the gait function of the rats with the postoperative period was found to improve using a filler concentration of 10 g/L compared to that of 14 g/L.

## 4. Discussion

The glucose/mannose used in this study has been acetylated to increase the water solubility. Due et al. have reported that acetyl groups in glucose/mannose affect water solubility and gelation [23]. Moreover, they have also clarified that increasing the concentration of glucose/mannose promotes a faster gelation rate and kinematic viscosity. The concentration of glucose/mannose is not affected by static viscosity and kinematic viscosity increases with increasing the concentration; that is, dynamic viscoelasticity has been reported to enhance with increasing the concentration [24]. In the present work, there was no significant difference in static viscosity between 10 and 14 g/L concentrations of glucose/mannose filler, but the kinematic viscosity at a concentration of 10 g/L was significantly lower than that at a concentration of 14 g/L. Since the glucose/mannose used as raw materials have the same molecular weight and backbone, the difference in kinematic viscosity of the glucose/mannose filler is supposed to be due to the concentration of glucose/mannose in the aqueous solution, i.e., the density of the solute in the solvent.

Adding alkali plays an important solubilizing role in the gelation of glucose/mannose and promotes the deacetylation of the chains. Deacetylation is important in progressively disabling the polyelectrolytic polysaccharide chains by alkali through the pH change induced by the reaction, reducing the inherent water solubility of the polymer [25]. In this study, to fabricate hollow glucose/mannose conduits, Na_2_CO_3_ was added as an alkali to the glucose/mannose aqueous solution, which was expected to deacetylate the chains. Wong et al. reported that the addition of Na_2_CO_3_ promoted the polymerization of glucose/mannose and formed an insoluble film [26]. In this study, hollow conduits of glucose/mannose were found at 4–12 weeks postoperative. Therefore, glucose/mannose is supposed to increase the degree of polymerization with the addition of Na_2_CO_3_, becoming insoluble nerve guidance conduits in vivo. The immersion of the hollow glucose/mannose conduits in distilled water caused a broadening of the XRD diffraction peaks. The intensity of these peaks also decreased upon immersion. Glucose/mannose is known to absorb and swell with large amounts of water [27]. Water molecules penetrate between the polymer chains of glucose/mannose and interact with the hydroxy groups of the glucose/mannose backbone. This interaction causes the polymer backbone to swell and become more disordered, possibly affecting the crystallinity of the glucose/mannose chain. The broadening and intensity decrease of these peaks may be due to the reduced crystallinity of glucose/mannose conduits with a layered structure. The interlayer distance is presumed to extend with water immersion because some of the water molecules were inserted between the layers. 

Mechanical properties play an important role in implementing constant structural support to protect the nascent axon from scar tissue invasion, prevent the compression of surrounding tissues against regenerative cells, and ensure successful nerve regeneration. Despite numerous reports on peripheral nerve stretch injuries [20,28,29,30,31], few studies have compared human nerve histology and mechanical properties. Kerns et al. have compared the tibial nerve following total hip arthroplasty and hip trauma using two nerves from fresh human cadavers with or without controlled stretch and revealed the mechanical properties of the human peroneal nerve, with Young’s modulus of 10.4 MPa and maximum tensile strength of 3.88 MPa [32]. In this work, Young’s modulus and the maximum tensile strength of hollow glucose/mannose guidance conduits were 16.92 ± 3.10 and 9.23 ± 0.91 MPa, respectively, which were higher than the human peroneal nerve. This resulting tensile strength and Young’s modulus can be expected to have the potential to reduce damage caused by nerve elongation during the period applied with the glucose/mannose nerve guidance conduits.

In the results of this study, nerve regeneration could be achieved in a 6 mm gap using the glucose/mannose nerve guidance conduits, and myelinated nerve and glial cell development and nerve fiber continuity became more pronounced with increasing postoperative period, regardless of kinematic viscosity. Nerve regeneration is associated with axonal growth. Axonal growth has been known to be driven by a motile growth cone that develops at the tip of a nerve injury [33,34]. Motile growth cones derive growing axons through developing tissues to their synaptic targets [35]. The continuous advance of the growth cone depends on the dynamic assembly, turnover, organization, etc., which is associated with adenosine triphosphate (ATP) as an energy source [36,37]. Even in this work that uses glucose/mannose nerve guidance conduits, nerve elongation should be supposed to originate from axonal growth based on the above-mentioned reports. Since ATP is produced in the glycolytic metabolic pathway, glucose/mannose as a filler in hollow guidance conduits may serve as an energy source for axonal growth. At 8 and 12 weeks postoperative, the glucose/mannose used as a filler remained slightly in the regenerated nerve tissue at a concentration of 14 g/L but not at a concentration of 10 g/L. The reason why glucose/mannose remained in the regenerated nerve tissue may be due to undigested glucose/mannose during the ATP generation process and/or uptake due to increased kinematic viscosity.

In statistical research on functional recovery after nerve repair, the following report has been published on neuroanastomosis in patients with nerve ruptures [2]; nerve regeneration using a medical nerve guidance conduit also had the same level of functional recovery as neuroanastomosis. On the other hand, Duan et al. assessed gait functional recovery at 4–12 weeks postoperative with the sciatic function index (SFI) using a rat model [38]. Takeya et al. evaluated a rat’s gait function recovery with SFI using the nerve guidance conduits consisting of an outer layer of chitosan hydrogel and an inner layer of Schwann cell-encapsulated collagen hydrogel [39]. From these reports, the recovery of motor function can be estimated to be 40–50% for autografts and 30–40% for nerve guidance conduits at 12 weeks postoperative. In this work based on the angle between the metatarsophalangeal joint and ground plane, the functional recovery corresponded to approximately 70% at 12 weeks postoperative using a filler with a glucose/mannose concentration of 10 g/L, resulting in superior gait functional recovery. The histological evaluation suggested that this recovery of gait function was reasonable, as nerve fibers, myelinated nerve, and glia cell expression approached normal tissue at 12 weeks postoperative. At a glucose/mannose concentration of 14 g/L, the gait function recovery corresponded to approximately 55% at the same period lower than at a glucose/mannose concentration of 10 g/L, probably due to adverse effects with glucose/mannose contamination within regenerated nerves. The easy comparison of the functional recovery reported in this study with those in other reports due to the different gait function assessments, length of nerve regeneration, conditions of stimulation applied to the nerve, etc., may be difficult, but the nerve guidance conduits consisting of glucose/mannose would have high efficacy for nerve regeneration and gait function recovery. 

## 5. Conclusions

The nerve regeneration and gait function recovery with the crystalline glucose/mannose nerve guidance conduits filled with glucose/mannose viscus solution were examined using the defect model of the sciatic nerve in a rat. The crystalline glucose/mannose nerve guidance conduits with hydrophilicity and surface flatness exhibited mechanical strength suitable for nerve regeneration. The sciatic nerve developed rapidly and regenerated into the 6 mm gap at 8 weeks postoperative, regardless of the glucose/mannose concentration as a filler. A glucose/mannose filler with a concentration of 10 g/L resulted in an excellent recovery of gait function at 12 weeks postoperative. The glucose/mannose used in this work was effective as a new candidate material for peripheral nerve regeneration.

## Figures and Tables

**Table 1:** The inspection results for safety standard values to humans of glucose/mannose used in this work.

	Safety Standard Value	Inspection Results
pH value	5~7	6.83
Protein	≤1.2%	0.2%
Lipid	≤0.2%	0.0%
Arsenic	No detection	No detection
Heavy metals	No detection	No detection
Sulfites	No detection	No detection
General bacteria	Negative	Negative
Coliform bacteria	Negative	Negative

## Data Availability

Data are contained within the article.
